# The genomic landscape of the immune system in lung cancer: present insights and continuing investigations

**DOI:** 10.3389/fgene.2024.1414487

**Published:** 2024-06-25

**Authors:** Mina Roshan-Zamir, Aida Khademolhosseini, Kavi Rajalingam, Abbas Ghaderi, Raja Rajalingam

**Affiliations:** ^1^ School of Medicine, Shiraz Institute for Cancer Research, Shiraz University of Medical Sciences, Shiraz, Iran; ^2^ Cowell College, University of California, Santa Cruz, Santa Cruz, CA, United States; ^3^ Department of Immunology, School of Medicine, Shiraz University of Medical Sciences, Shiraz, Iran; ^4^ Immunogenetics and Transplantation Laboratory, University of California San Francisco, San Francisco, CA, United States

**Keywords:** lung neoplasms, immunogenetics, immunotherapy, genetic susceptibility, genetic variation

## Abstract

Lung cancer is one of the most prevalent malignancies worldwide, contributing to over a million cancer-related deaths annually. Despite extensive research investigating the genetic factors associated with lung cancer susceptibility and prognosis, few studies have explored genetic predispositions regarding the immune system. This review discusses the most recent genomic findings related to the susceptibility to or protection against lung cancer, patient survival, and therapeutic responses. The results demonstrated the effect of immunogenetic variations in immune system-related genes associated with innate and adaptive immune responses, cytokine, and chemokine secretions, and signaling pathways. These genetic diversities may affect the crosstalk between tumor and immune cells within the tumor microenvironment, influencing cancer progression, invasion, and prognosis. Given the considerable variability in the individual immunegenomics profiles, future studies should prioritize large-scale analyses to identify potential genetic variations associated with lung cancer using highthroughput technologies across different populations. This approach will provide further information for predicting response to targeted therapy and promotes the development of new measures for individualized cancer treatment.

## 1 Introduction

Lung cancer is the primary cause of cancer-related deaths worldwide, accounting for 1.7 million deaths and 2.2 million new cases in 2020, with projections indicating an increase to 3.5 million cases by 2040 (International Agency for Research on Cancer, 2020). It is categorized into two main subgroups, small cell lung cancer (SCLC) and non-small cell lung cancer (NSCLC), accounting for 10%–15% and 80% of all cases, respectively. SCLC can be histologically subdivided into small cell carcinoma, mixed small cell/large cell cancer, and combined small cell lung cancer. On th other hand, NSCLC is classified into lung adenocarcinoma (LUAD), lung squamous cell carcinoma (LUSC), and large-cell carcinoma (LCC), representing the most common subtypes (Wu et al., 2023). Smoking is widely recognized as the leading cause of lung cancer, with approximately 90% of cases attributed to tobacco consumption (Alberg and Samet, 2003). Moreover, several factors such as age (typically over 65 years), sex, and environmental and genetic factors influence the development of lung cancer (Cheng et al., 2021; Huang et al., 2021). For instance, genetic alterations in oncogenic drivers, such as the Kirsten rat sarcoma (*KRAS*), v-raf murine sarcoma viral oncogene homolog B (*BRAF*), and epidermal growth factor receptor (*EGFR*) genes, are commonly found in patients with NSCLC and can affect their response to treatments (Chevallier et al., 2021). Owing to the absence of typical early symptoms and effective early detection strategies, approximately 70% of lung cancer patients are diagnosed at an advanced-stage or with distant metastasis, leading to a 5-year survival rate of only about 4% (Ren et al., 2021; Wu et al., 2023). Three common treatment options for lung cancer include surgery, chemotherapy, and radiotherapy. Recently, immune checkpoint inhibitors, such as antibodies against programmed death-1 (PD-1), programmed death-ligand 1 (PD-L1), and cytotoxic T lymphocyte-associated antigen 4 (CTLA-4), have greatly improved cancer treatment. However, the response to these therapies is still relatively low (Lahiri et al., 2023).

The immune system plays a significant role in monitoring, recognizing, and destroying tumor cells under homeostatic conditions. Therefore, any dysregulation in immune-related responses and genomic-level mutations should be scrutinized as they may be related to lung cancer pathogenesis (Smok-Kalwat et al., 2023). The lung tumor microenvironment (TME), characterized by a heterogeneous cell population and intricate interactions, plays a significant role in the progression of lung cancer and the efficacy of treatment (Zhang et al., 2021). In recent years, the development of novel techniques, such as single-cell RNA sequencing (scRNA-seq), has enabled scientists to identify genetic and epigenetic changes that may increase lung cancer risk, as well as different aspects of the disease, such as its TME, at the genomic and transcriptomic levels. Furthermore, these advancements have improved personalized immunotherapies and the identification of individuals who can benefit more from specific treatments based on their genetic backgrounds. For instance, investigating single-cell RNA-Seq data from patients with LUAD and cell lines revealed heterogeneous expression of major histocompatibility complex (*MHC*) *class II* genes and significant downregulation of interferon (*IFN*)*-γ* signaling genes. This downregulation was found to be associated with immunotherapy resistance (Ma et al., 2019). Despite previous investigations, there is a growing need to identify immune response-related genes that may be associated with the susceptibility, treatment response, and prognosis of lung cancer.

The primary cause of death in patients with lung cancer is recurrence after chemotherapy or surgical intervention (Uramoto and Tanaka, 2014). Therefore, identifying novel biomarkers for early detection and developing therapeutic approaches based on immunogenic markers to reduce metastasis and enhance overall survival in patients with lung cancer is crucial. This review aimed to investigate the potential association between genetic variations in innate and adaptive immune cells, cytokines, and chemokines and susceptibility, prognosis, and response to immunotherapy in patients with lung cancer.

## 2 Tumor mutational burden

The TMB is characterized by the number of somatic coding mutations per megabase in the tumor genome and has emerged as a potential predictor of immunotherapy response in various tumor types (McNamara et al., 2020). The correlation between TMB and tumor immunology is complex and requires further investigation. Tumors with increased TMB often show higher expression of cancer-specific antigens, known as neoantigens. These somatic, non-synonymous mutations can lead to the expression of immunogenic epitopes that are specifically found in cancer cells (Chae et al., 2018; Wang P. et al., 2021). Lung cancers are among the malignancies with high TMB (Chalmers et al., 2017). An analysis of data from patients with LUAD in the Cancer Genome Atlas (TCGA) database revealed that elevated TMB is associated with increased infiltration of various immune cell populations, favoring an anti-tumor immune response. Tumors harboring mutations in DNA repair-associated genes, including mismatch repair genes, homologous recombination genes, and polymerase epsilon (*POLE*) exhibit significantly higher mutational counts, neoantigen quantity, and T cell infiltration (Chae et al., 2018). Research has also demonstrated an association between high mutational load and the overexpression of immune-related genes, such as interleukin-12 receptor subunit beta-2 (*IL12RB2*) and *IFN-γ*, which are related to Th1 anti-tumor reponse and *IL-21*, *IL-23A*, and interleukin 17 receptor A (*IL17RA*), which play roles in Th17-related pathways (Ye et al., 2013; Chae et al., 2018). Th17 may have both pro- and anti-tumorigenic roles in cancer. In contrast, cytokine genes associated with Th2 response such as *IL-33* and thymic stromal lymphopoietin (*TSLP*) have shown an inverse correlation with neoantigen burden. High neoantigen load was also correlated with the overexpression of genes related to the cytotoxic function of CD8^+^ T cells, including granzyme B (*GZMB*) and FAS ligand (*FASLG*), as well as *TAP2* which facilitates antigen processing in the MHC I antigen presentation pathway. Additionally, higher mutational load has been positively correlated with the overexpression of the chemokines tumor necrosis factor receptor superfamily member 25 (*TNFRSF25*), C-C chemokine receptor type 1 (*CCR1*), and lymphotoxin beta receptor (*LTBR*) in patients with LUAD. Neoantigens likely promote M1 macrophage polarization, enhancing anti-tumor immunity by increasing interactions between Th1/Th17 cell responses and other innate immune cells. In this regard, a higher mutational load was correlated with both higher and lower expression levels of several M1 and M2 macrophage-associated genes, respectively (Chae et al., 2018). In patients with NSCLC treated with immune checkpoint inhibitors, responders with the highest TMB were found to have mutations in genes associated with DNA repair and replication, including the polymerase delta 1 catalytic subunit (*POLD1*), *POLE*, and MutS homolog 2 (*MSH2*) (Chae et al., 2018).

Several studies have reported a higher TMB in patients with a history of smoking (Xiao et al., 2017; Chae et al., 2018). Moreover, patients of East Asian ancestry with LUAD have less genomic complexity, characterized by fewer mutations and copy number alterations compared to those of European ancestry (Chen et al., 2020).

In patients with NSCLC undergoing treatment with immune checkpoint inhibitors, the number of neoantigens per tumor is correlated with the mutation burden. Tumors from patients who experience sustained clinical benefits showed a remarkably higher neoantigen burden compared to those from individuals without durable benefit. Moreover, an increased neoantigen burden is associated with improved progression-free survival (Rizvi et al., 2015). For patients with NSCLC receiving treatment with immune checkpoint inhibitors, either as monotherapy or in combination, higher levels of TMB have been associated with positive outcomes, such as improved objective response, durable clinical benefit, and prolonged progression-free and overall survival (Rizvi et al., 2015; Hellmann et al., 2018; Rizvi et al., 2018).

In summary, analyzing TMB represents progress in the search for biomarkers of lung cancer. Whole-exome sequencing (WES) with next-generation sequencing (NGS) stands as the gold-standard method for evaluating tissue TMB. However, several limitations such as high cost, prolonged turnaround time, large input DNA requirements, and technological complexity make it impractical for routine clinical practice (Berland et al., 2019). Consequently, targeted gene panels have emerged as viable alternatives in clinical settings, demonstrating a strong correlation with the WES platform (Hellmann et al., 2018; Rizvi et al., 2018). Yet, the diversity in methods and criteria used to assessTMB necessitates the standardization of key aspects of panel-based TMB estimation. Therefore, it is crucial to conduct future clinical trials to confirm TMB as a reliable biomarker and improve its efficacy in guiding clinical decisions.

## 3 Innate immunity

### 3.1 Neutrophils

Chronic obstructive pulmonary disease (COPD) is characterized by chronic inflammation affecting the lung parenchyma and airways, resulting in irreversible and progressive restriction of airflow. There is a strong association between COPD and the development of lung cancer, as individuals diagnosed with COPD are more likely to develop lung cancer (Young and Hopkins, 2018). Neutrophilic inflammation has been identified as a key factor in the progression of COPD (Aloe et al., 2021). Studies have been conducted to identify neutrophil biomarkers that can assist in predicting the prognosis of NSCLC patients and their response to immunotherapies. Integrated data from scRNA-seq and bulk RNA-seq have suggested a prognostic risk model that includes six neutrophil differentiation-related genes based on the rate of four reference genes (*ACTB*, *GAPDH*, *TFRC*, *TUBB*). The identified genes are *MS4A7*, *CXCR2*, *CSRNP1*, *RETN*, *CD177*, and *LUCAT1*, all of which are primarily associated with immune-related pathways (Pang et al., 2022). Additionally, depletion of Toll-like Receptor (*TLR*) *2* and *TLR4* resulted in a significant decrease in the proportion of neutrophils and their related cytokines in tumor tissues and bronchoalveolar lavage fluid (BALF), respectively (Jungnickel et al., 2017). In the case of cancer, precancerous cells can be activated through endogenous TLR ligands secreted from inflammatory cells, leading to the overexpression of angiogenic factors, cytokines, and growth factors. Therefore, TLRs can create a microenvironment favorable for tumor progression (He et al., 2007; Szajnik et al., 2009; Wang et al., 2010). A significant correlation has been observed between the *TLR4* rs4986791 (+1196C/T) gene polymorphism and the risk of lung cancer. This correlation is likely due to the effect of functional polymorphisms on modulating inflammation mechanisms (Kurt et al., 2016). Moreover, the *TLR4* rs7869402C > T polymorphism was found to be associated with a higher risk of NSCLC. This SNP is located in the 3′untranslated region of *TLR4*, which affects its binding to miRNA and subsequently impacts mRNA expression (Wu et al., 2020).

Studies using lung tumor IL-17:Kras^G12D^ murine models have suggested the tumor-promoting role of the IL-17-neutrophil axis, as IL-17A increased the infiltration of neutrophils to the tumor site. These mice were also found to be resistant to treatment with PD-1 blockade (Akbay et al., 2017). Studies have also indicated an association between various polymorphisms in the *IL-17A* gene and an elevated risk of lung cancer (Ma et al., 2015; He et al., 2017). A higher frequency of *IL-17A* rs8193037GA and AA genotypes has been linked to a predisposition to NSCLC, as having the rs8193037A allele was associated with higher production of IL-17 (Cheng et al., 2015a). Moreover, patients carrying the T-allele of *IL-17A* rs8193036(C > T) had a higher risk of developing advanced stages of LUAD than those having the CC genotype of rs8193036, probably through inducing the overexpression of IL-17A (Lee et al., 2021). These studies suggest that polymorphisms that cause IL-17 overexpression are associated with increased susceptibility and enhanced lung tumor progression. However evaluating the expression level of IL-17A and checking the polymorphisms in different populations are required to validate the findings.

### 3.2 Natural killer cells

Under steady-state conditions, the lung environment must be carefully regulated to tolerate environmental antigens and commensal bacteria (Culley, 2009). Studies have demonstrated that lung NK cells are more mature (CD11^brigh^ CD27^low^), express lower levels of activating co-stimulatory molecules (such as CD1d, CD86, and B220), and express higher levels of inhibitory receptors (CD94-NKG2A) than activating receptors (NKG2D) compared with those in the bone marrow or spleen, which helps maintain pulmonary homeostasis (Wang et al., 2012). The final activity of NK cells is determined by the signals from various inhibitory or activating receptors (Djaoud and Parham, 2020). Killer-cell immunoglobulin-like receptors (KIR) are germ-line encoded receptors that bind to classical human leukocyte antigen (HLA) class I molecules as their ligands, a process named “licensing,” which plays an important role in NK cell development. *KIR* and *HLA* loci show considerable variation among populations (Pyo et al., 2010). A recent study in southern Iran revealed an association between specific *KIR* gene clusters and *HLA* ligands, with susceptibility to lung cancer. Higher frequencies of *KIR2DL2* and *KIR2DS2* have been reported in patients with lung cancer, individually and in combination with their ligand (*HLA-C1*), compared with healthy controls. This susceptibility could be the result of impaired missing self-recognition in carriers of *KIR2DL2/HLA-C1* with lung cancer tumors sustaining HLA-I expression or from hypo-responsiveness owing to KIR2DS2/HLA-C1 interactions. In contrast, higher frequencies of *KIR2DS1* and *KIR3DS1* genes probably conferred protection against this cancer, as these NK cells have a lower activation threshold, allowing for easier activation in the absence of their respective ligands. Patients with lung cancer also demonstrated higher levels of inhibitory *KIR* genes than activating genes. The presence of an increased number of inhibitory *KIR* genes can attenuate NK cell function in lung tumor cells, facilitating tumor evasion within the suppressive TME of lung cancer (Hematian et al., 2022). Further studies have also reported the association of rs1639113 and rs9265821 polymorphisms in the 3’ region of the *HLA-C* and *HLA-B* genes with the risk of lung cancer, respectively. These results were based on a genome-wide association study (GWAS) analysis in a Croatian population consisting of 203 patients with lung cancer and 3,60,938 controls (Baranasic et al., 2023). The *HLA* genotype affects which neoantigen, arising from tumor cell mutations, will be represented to the immune system and stimulate the subsequent elimination of tumor cells. Therefore, mutations that give rise to neoantigens with poor representation by HLA molecules or the downregulation of neoantigen-presenting HLA-I molecules by the tumors, such as NSCLC, can help them evade anti-tumor responses (Perea et al., 2017; Saab et al., 2020). Reduced expression of HLA-I molecules can result from *HLA* haplotype loss or the downregulation of *HLA* genes, such as *β2-microglobulin* (*B2M*) or antigen processing machinery genes such as *TAP1/2*, *Tapasin*, and *LMP2/7* (Perea et al., 2017). Further investigations have shown that HLA loss of heterozygosity (LOH) is an immune escape mechanism in approximately 40% of early-stage NSCLC and is associated with an increase in the frequency of sub clonal mutations and neoantigen burden. Tumors with high HLA LOH tend to have more neoantigens that are predicted to bind to the lost HLA molecules, making these neoantigens invisible to the immune system. Additionally, tumors with high HLA LOH showed increased PD-L1 expression in immune cells and RNA signatures that suggest immune activation. Overall, the consideration of HLA LOH allows for the prediction of a set of neoantigens that can potentially stimulate T cell responses more effectively. This understanding may be beneficial for the development of future neoantigen-based immunotherapies (McGranahan et al., 2017). In this context, *in silico* immunogenomic tools are being developed to predict the HLA class I-binding affinity for each tumor-specific set of peptides, including neoantigens. The stability of these interactions strongly correlates with T cell immunogenicity (Liu and Mardis, 2017). In the case of other NK cell ligands, an *in silico* study by Kucuk et al. demonstrated decreased expression of several members of the NKG2D ligands in the LUAD group compared with the normal group. This downregulation is considered an anti-tumor escape mechanism, with MHC class I polypeptide-related sequence A (MICA) showing the most significant decrease. Lower expression of MICA transcripts may also be regarded as a prognostic factor for LUAD. Furthermore, MICB transcript expression correlates with immune cell infiltration (Kucuk and Cacan, 2022). These findings suggest that genes encoding NK cell receptors and their ligands can be valuable targets for predicting susceptibility to lung cancer, offering prognostic value and potential therapeutic targets in future immunotherapies.

In addition to NK cell receptors and their ligands, mutations in several other genes can affect NK cell function in lung cancer. For instance, higher expression of erythropoietin-producing hepatocellular receptor A5 (EPHA5), a member of the Eph family of tyrosine kinase receptors, is associated with lymph node metastasis, TNM stage, and LUAD differentiation (Liu et al., 2022). In a recent study by Li et al., the overexpression of wild-type *EPHA5* suppressed tumor invasion and migration, in contrast to the effects observed with mutant *EPHA5*. Further analysis demonstrated that overexpression of wild-type *EPHA5* significantly promoted NK cell proliferation and cytotoxicity against NSCLC cells and was associated with decreased cell apoptosis. However, mutant EPHA5 impairs NK cell activity and enhances the migration and invasion of NSCLC cells (Zhang et al., 2020). Fatty acid-binding protein (FABP) 5 regulates lipid metabolism and is overexpressed in various cancers, including lung cancer, with high expression associated with poor prognosis (George Warren et al., 2023). To elucidate the potential association between lipid homeostasis and lung cancer metastasis, the levels of IFN-γ and granzyme B produced by lung NK cells in tumor-bearing *Fabp5* ^−/−^ mice were significantly decreased compared with their wild-type counterparts, suggesting a role for *FABP5* in regulating the cytotoxicity of NK cells. Moreover, the maturation of lung-derived NK cells decreased in tumor-bearing *Fabp5* ^−/−^ mice, likely due to the reduced expression of T-bet and Eomes transcription factors. These events lead to enhanced lung cancer metastasis (Yang et al., 2021). Recent studies have also indicated that apolipoprotein E (ApoE), which is involved in cholesterol metabolism, is overexpressed in human lung cancer tissues compared with adjacent non-cancerous tissues (Trost et al., 2008). This overexpression is associated with a poor prognosis due to the promotion of tumor progression and invasion in patients with LUAD (Su et al., 2011). Further investigations have demonstrated that inhibition of *ApoE* enhances NK cell cytotoxicity and increases NK cell infiltration by upregulating triggering receptors expressed on myeloid cells (TREM)-1 and T-bet (Lee et al., 2019a). Higher expression of APOE has been reported in macrophages within tumor tissues than in normal tissues (He et al., 2021). Serine/threonine kinase 11 (*STK11*) is a tumor suppressor gene, and loss-of-function mutations in this gene have been associated with enhanced tumor escape, invasion, and metastasis (Mazzaschi et al., 2021). These mutations can affect the function of immune cells and change the composition of the TME. For example, *STK11* deletion significantly reduces the cytotoxicity of NK cells and their infiltration into the TME, ultimately promoting the proliferation of LUAD cells (Huang et al., 2022). Consistent with previous research, the overexpression of AXL, a member of the TAM (Tyro3, Axl, and Mer) receptor tyrosine kinase family, in mesenchymal carcinoma cells likely plays a role in the cell-intrinsic immune escape mechanism in NSCLC patients by suppressing the killing effects of NK cells and cytotoxic T lymphocytes (CTLs). Consequently, targeting AXL is a potential strategy for sensitizing mesenchymal NSCLC clones to lymphocyte-mediated cytotoxicity. AXL targeting induces NK-kB activation, resulting in enhanced expression of intercellular adhesion molecule 1 (*ICAM1*) and UL16 binding protein 1 (*ULBP1*) along with MAPK inhibition, finally improving the survival of patients with NSCLC. ULBP1 serves as a ligand for NKG2D, an activating receptor on NK cells, and its overexpression facilitates the recognition of stressed cells by the immune system. Therefore, one of the immune escape mechanisms involving AXL in carcinoma cells might be by decreasing the expression of ULBP1 (Terry et al., 2019). Further studies have also revealed the overexpression of the pancreatic progenitor cell differentiation and proliferation factor (PPDPF) in NSCLC tissue and cell lines, which is associated with lower patient survival and increased resistance of lung tumor cells to radiotherapy (Yun et al., 2022). Zheng et al. reported that the PPDPF-induced activation of STAT3 plays a significant role in decreasing NK cell activation and infiltration, creating an immunosuppressive microenvironment in LUAD (Zheng et al., 2022).

Recent investigations using advanced techniques such as scRNA-seq have led to the development of novel gene signatures derived from marker genes of tumor-infiltrating NK cells to predict the prognosis and response to immunotherapy in patients with lung cancer. For instance, Song et al. identified a novel seven-gene signature, including *GCSAML*, *ACTG1*, *ACOT7*, *SELENOK*, *PEBP1*, *BIRC3*, and *ACAP1*. These genes are primarily involved in the modulation of cell proliferation, migration, regulation of the cell cycle and NK cell functions in patients with LUAD (Song et al., 2022). A recent *in silico* study also suggested an antigen-presenting cell (APC)/T/NK cell-related gene signature consisting of 16 genes to estimate overall survival in patients with LUAD, which were mostly associated with immune-related pathways and responses. Three genes (*NCR3*, *RAET1E*, and *SHC1*) were closely linked to NK cell cytotoxicity pathways, while another set of three genes (*MAP2K1*, *NRAS*, and *PTPN6*) showed a significant association with T cell and NK cell-related pathways (Huang L. et al., 2023).

Investigation of microRNA editing levels has been suggested as a potential biomarker of LUAD (Maemura et al., 2018). MiRNAs affect the function, survival, and cytotoxicity of NK cells by regulating cytokine production (Shirshev et al., 2017). For instance, miR-218-5p impairs IL-2-induced NK cell cytotoxic capacity against LUAD cells by regulating the expression of serine hydroxymethyl transferase 1 (SHMT1), both *in vitro* and *in vivo* (Yang et al., 2019). Zhou et al. demonstrated elevated STAT3 mRNA levels and downregulated miR-130a levels in primary NK cells derived from patients with NSCLC compared with healthy controls. However, miR-130a overexpression in the IL-2-induced NK-92 cell line markedly increased cytokine production and the killing effect of NK cells on A549 cells by affecting STAT3 expression (Zhou et al., 2020). Consistent with previous research on NSCLC, the overexpression of hsa-miR-301a-3p can reduce the cytotoxicity of NK cells and decrease the secretion of IFN-γ and TNF by targeting Runt-related transcription factor 3 (RUNX3). This mechanism has also been shown to promote tumor progression in *in vivo* models (Zhang et al., 2023). A study conducted by Gao et al. also found decreased expression of miR-30c in primary NK cells from lung cancer patients compared to healthy controls. Overexpression of miR-30c increased NK cell cytotoxicity against A549 cells, while downregulation of miR-30c reduced the secretion of IFN-γ and TNF by these cells. The regulation of NK cell cytotoxicity by miR-30c occurs through targeting N-acetylgalactosaminyltransferase 7 (GALNT7) (Gao et al., 2023), a glycosylation enzyme that has oncogenic effects by binding to various miRNAs in different types of cancers (Nie et al., 2016; Wang JB. et al., 2020; Cao et al., 2020). Moreover, as GALNT7 mediates the phosphorylation of PI3K/AKT signaling, and its inhibition ultimately leads to the inactivation of this pathway, enhancing the killing effects of NK cells (Gao et al., 2023). These studies suggest that miRNAs play a role in regulating NK cell function and lung cancer immunopathogenesis, which could be considered in future NK cell-based anti-tumor immunotherapies. Long non-coding RNAs (lncRNAs) have also been implicated as regulators of immune cell differentiation and infiltration into tumors. *In silico* studies have suggested that several lncRNAs could be associated with immune-related pathways, specifically cytokines and cytokine receptors, as well as their valuable roles in classifying various cancer subtypes based on molecular and immunological features (Li Y. et al., 2020). A recent study using a computational approach has also revealed the significance of lncRNAs in predicting the response to immune checkpoint inhibitor immunotherapy in patients with NSCLC (Sun et al., 2020). Other studies have also investigated the anti-tumor functions of TME-derived lncRNAs in LUAD tumors. The *AC008750*.*1* non-coding transcript shows high expression in NK cells and CD8^+^ T cells and is positively correlated with NK cell granule protein 7 (*NKG7*) expression under stimulatory conditions, a marker associated with the granulation of NK cells and CD8^+^ T cells. To validate this, *AC008750*.*1* knockdown using siRNA significantly decreased the cytotoxicity of NK cells against LUAD cells (Sage et al., 2020). However, further research is necessary to enhance our comprehension of the roles of lncRNAs in modulating immune-related pathways, thereby improving patient responsiveness to immunotherapies.

### 3.3 Dendritic cells

Research findings indicate that lung tumor-infiltrating DCs express higher levels of CD11b and the inhibitory molecule PD-L1 than peritumoral lung DCs (Pyfferoen et al., 2017). Additionally, lung tumor-derived cDC1s express lower levels of T cell immunoglobulin mucin-4 (TIM4), leading to decreased tumor-associated antigen uptake and lower activation of CD8^+^ cells in advanced stages of murine lung tumors (Caronni et al., 2021). NSCLC-derived DCs demonstrate increased secretion of IL-10 and higher expression of B7-H3 T cell co-inhibitory molecules compared with their counterparts in normal lungs, creating an immunosuppressive TME where anti-tumor T cells fail to become effectively activated (Schneider et al., 2011). An immunosuppressive TME may be associated with a higher frequency of plasmacytoid DCs (pDCs) derived from NSCLC tissues, which are characterized by elevated expression levels of CD33 and PD-L1. Tumor-derived pDCs also reduce the production of type I IFNs (Pedroza-Gonzalez et al., 2015; Sorrentino et al., 2015). Previous investigations have indicated that increased secretion of transforming growth factor-beta (TGF-β) by DCs in patients with NSCLC is associated with higher levels of Tregs (Dumitriu et al., 2009), which can further contribute to the development of an immunosuppressive TME in NSCLC (Zhong et al., 2021). Dysfunction of tumor-induced DC in NSCLC may be caused by the inhibition of nuclear factor-kappa B (NF-kB) and signal transducer and activator of transcription 3 (STAT3) signaling pathways, leading to downregulation of downstream genes associated with cytokine and chemokine production, as well as antigen presentation (Li et al., 2017). Overall, DCs derived from lung cancer showed increased expression of genes incolved in creating an immunosuppressive TME, which promotes tumor progression.

Exploring the role of miRNAs in regulating the function of DCs adds another layer of complexity. *In silico* studies have revealed a potential association between the overexpression of miR-582, reduced expression of *CD1B*, and lower overall survival in advanced stages of lung cancer. This miRNA regulates *CD1B*, a marker of resting and activated DCs engaged in the presentation of lipids and glycolipids to T cells via the MHC complex. Identifying potential miRNA biomarkers implicated in regulating immune cell genes, particularly their target immune genes, holds promise for identifying novel immunotherapies to impede tumor progression in patients with lung cancer. Manipulation of the expression of these miRNAs is achievable through interventions, such as small interfering RNA, miRNA mimics, and small molecule inhibitors of miRNAs. However, experiments with larger sample sizes, evaluation of expression levels in tumor tissues and their adjacent counterparts, and further experimental methodologies are required to confirm these findings (Guo et al., 2020).

These findings suggest that changes in the phenotype and function of DCs in lung tumors contribute to an immunosuppressive microenvironment, hindering effective T cell activation. These alterations may be considered potential immune evasion mechanisms in lung cancer cells. Understanding the molecular mechanisms underlying DC dysfunction in lung tumors will enable us to target and manipulate these inhibitory mechanisms, ultimately improving the overall survival of patients with lung cancer.

### 3.4 Macrophages

Tumor-associated macrophages (TAMs) exhibit distinct phenotypes known as inflammatory or classically activated (M1) and anti-inflammatory or alternatively activated (M2) macrophages. The M1 phenotype is associated with both pro- and anti-tumorigenic roles, whereas the M2 phenotype is characterized by tumor-promoting properties. This duality plays a significant role in the cancer immune microenvironment, influencing the growth, progression, and metastasis of lung tumors (Sedighzadeh et al., 2021). M2-like TAMs play a crucial pro-tumor role in lung cancer. The observed decrease in M2-like TAM population within the TME impedes lung cancer growth and metastasis (Kawaguchi et al., 2023). Tumor cells play an important role in M2 phenotype polarization in the TME by releasing cytokines. For instance, IL-37 was found to induce the expression of a scavenger receptor, known as the macrophage receptor with a collagenous structure (MARCO), on TAMs. MARCO^+^ TAMs are anti-inflammatory macrophages with pro-tumoral effects in the TME (Georgoudaki et al., 2016). Depletion of the *IL-37* gene in two distinct lung cancer cell lines (H460 and A549) suppressed MARCO expression and diminished IL-10 production in co-cultured macrophages (La Fleur et al., 2021). Moreover, analysis of RNA-seq data from patients with NSCLC revealed a positive correlation between the gene expression of MARCO and the expression of various genes associated with immunosuppressive TAMs, including CD68, CD163, macrophage scavenger receptor 1 (MSR1), IL4R, TGFB1, and IL10 (La Fleur et al., 2018). IL-17D is another cytokine that facilitates the infiltration of TAM in lung cancer cells. The induction of *IL-17D* expression in LLC1 cells, which typically lack this gene, resulted in a significant increase in F4/80^+^ CD11b^+^ TAMs in a subcutaneous tumor model. M2 macrophages (CD206^+^ F4/80^+^ CD11b^+^ TAMs) within total TAMs were prominently elevated in these tumors. Induction of IL-17D expression in A549 cells, which also do not endogenously express *IL-17D*, led to elevated expression of macrophage-related genes such as *CCL3*, *CCL4*, and *CSF1*. Further investigations using an *IL-17D* knockdown lung cancer cell line (H1155) revealed that IL-17D upregulates the genes related to macrophage-recruitment and polarization through the p38 MAPK signaling pathway (Lin et al., 2022).

Another recognized gene linked to M2 macrophage polarization is the one encoding Mincle (Li C. et al., 2020). Mincle or macrophage-inducible C-type lectin (Clec4e) is a transmembrane pattern recognition receptor expressed in myeloid cells (Huang X. et al., 2023). The majority of CD163^+^ cells in NSCLC are Mincle^+^ TAMs. Silencing *Mincle* in bone marrow-derived macrophages (BMDM) significantly increased the expression of M1 markers (iNOS, MCP-1, and TNF) upon stimulation with LLC conditioned medium. Analysis of scRNA-seq data from patients with NSCLC revealed a significant association between Mincle expression in the stroma and tumor, and unfavorable disease-specific survival (Li C. et al., 2020). Clustering of the immune components of LUAD samples in The TCGA database identified the gene encoding nucleotide-binding oligomerization domain-containing protein 2 (NOD2) as an important gene with tumor-suppressing effects. Lower levels of NOD2 expression are associated with unfavorable characteristics, such as larger tumor size, metastatic tumors, and advanced stages. *NOD2* knockdown in a human monocytic cell line (THP-1) resulted in decreased expression of M1 markers under lipopolysaccharide stimulation. However, M2 markers increased upon stimulation with IL‐4. Therefore, NOD2 deficiency was identified as a driving factor in the induction of protumorigenic macrophages in LUAD (Wang Y. et al., 2021).

The gene encoding estrogen receptor α (ERα) is another identified gene associated with macrophage infiltration and polarization in lung cancer. The expression of ERα has been linked to a poor prognosis in NSCLC (Castellanos et al., 2023). Inducing the overexpression of ERα in NSCLC cells resulted in increased recruitment of macrophages and induced M2 polarization. Conversely, downregulation of ERα using ERα‐shRNAs significantly reduced macrophage infiltration and induced M1 polarization. Further investigations revealed that ERα activates the CCL2/CCR2 axis, facilitating macrophage infiltration, M2 polarization, and MMP9 production, thereby enhancing the invasiveness of the NSCLC cells (He et al., 2020). Analysis of mRNA-seq data obtained from the TCGA database indicated that metastasis-associated gene 1 (*MTA1*), an oncogene involved in NSCLC metastasis, is associated with macrophage infiltration and the malignant phenotype of lung cancer. Moreover, the positive association of *MTA1* with CD206 in NSCLC and LUSC suggests the involvement of this gene in the infiltration of the protumorigenic phenotype of macrophages into the TME (Ma et al., 2022).

Studies have identified transcription factors involved in macrophage polarization in lung cancer. One such transcription factor is Krüppel-like factor 4 (KLF4), which plays a role in macrophage infiltration and polarization in NSCLC. KLF4 is expressed at higher levels in M2 macrophages compared to M0 macrophages (Arora et al., 2021). Furthermore, research in *c-Maf* knockout mice has confirmed the role of this transcription factor in controling M2-related genes. TAMs in the LLC mouse model show elevated levels of c-Maf expression. When *c-Maf* was silenced in TAMs, there was a decrease in the expression of *Il10*, *Arg1*, indoleamine 2,3-dioxygenase (*Ido*), and *Vegfa*, along with an increase in the levels of *Il12* and *Tnf*. Moreover, *c-Maf* knockdown TAMs significantly enhanced the production of IFN-γ from CD4^+^ and CD8^+^ T cells. Moreover, inducing LCC in mice with myeloid-specific genetic ablation of c-Maf resulted in a significantly lower tumor burden and smaller tumor size. Notably, c-Maf chromatin immunoprecipitation sequencing indicated that *c-Maf* directly regulates the colony-stimulating factor 1 receptor (*CSF1R*) locus in M2 BMDMs (Liu et al., 2020). CSF1, also known as macrophage colony-stimulating factor (M-CSF), and its receptor (CSF1R) play an important role in the polarization of M2 macrophage (Valero et al., 2021). CSF-1R^+^ macrophages are related to immunosuppression and tumor progression, and the expression of CSF-1R in TAMs is associated with an unfavorable prognosis and resistance to tumor immunotherapy (Koh et al., 2014; Candido et al., 2018; Gyori et al., 2018).

Regarding the implicated signaling pathways, the β–catenin signaling has emerged as a critical pathway in the transition of macrophages from M1 to M2 in lung cancer. The induction of lung cancer in mice with a specific knockout of β-catenin in macrophages led to a significant decrease in tumor growth compared to their wild-type counterparts. TAM analysis of these transgenic mice revealed an increase in the expression of M1 markers and a concomitant reduction in the expression of M2 markers (Sarode et al., 2020a). Additionally, inhibition of Wnt/β-catenin signaling with the molecular inhibitor of Wnt secretion, LGK-974, increased M1 markers and decreased M2 markers in NSCLC cells (A549 and H1299) (Tang et al., 2021). Moreover, the TGF-β1/β-catenin pathway in tumor cells and TAMs was observed to be involved in SCLC pathogenesis (Ahirwar et al., 2023). In gene silencing/upregulating studies, different genes have been discovered downstream of the β-catenin pathway. BMDMs isolated from *β-catenin* knockout mice showed significant downregulation of FOS-like antigen 2 (FOSL2) and upregulation of the AT-rich interaction domain 5A (ARID5A). FOSL2 and ARID5A have been identified as downstream transcription factors that are crucial for orchestrating the M1 to M2 transition (Sarode et al., 2020a). Furthermore, it was discovered that slit guidance ligand 2 (SLIT2) and its receptor, roundabout guidance receptor 1 (ROBO1), are downstream of the TGF-β1/β-catenin pathway. *SLIT2* has been identified as a tumor suppressor gene in SCLC, with its expression significantly reduced in this type of tumor, while *ROBO1* expression is significantly higher in SCLC cells compared to adjacent normal cells (Peifer et al., 2012; Ahirwar et al., 2023). Overexpression of *SLIT2* and deletion of *ROBO1* in SCLC cells have shown the anti-tumor and pro-tumor effects of SLIT2 and ROBO1, respectively, through macrophage polarization (Ahirwar et al., 2023).

M2 macrophages play a crucial role in promoting NSCLC metastasis. Guo et al. identified αB-crystallin (CRYAB) as a key factor in this process (Guo et al., 2019). CRYAB belongs to the small heat shock protein family and is actively involved in various signaling pathways, including apoptosis, inflammation, and oxidative stress (Zhang et al., 2019). When *CRYAB*-knockout NSCLC cell lines were co-cultured with M2 macrophages, a significant decrease in cell invasion and the epithelial-to-mesenchymal transition was observed. This effect was attributed to the CRYAB-mediated activation of the ERK1/2/Fra-1/Slug pathway (Guo et al., 2019).

Several lncRNAs are involved in macrophage polarization in lung cancer. These lncRNAs, known for their widespread expression, play pivotal roles in the regulation of gene expression. *LOC100270746* is a lncRNA located on chromosome 6p22.2. The expression of *LOC100270746* was discovered to be lower in LUAD tissues than in normal lung tissues. Low levels of *LOC100270746* were associated with unfavorable features, such as larger tumor size, local invasion, advanced TNM stages, and poor overall survival. Silencing *LOC100270746* with shRNA in a LUAD cell line (HCC827) revealed the anti-tumor effects of this lncRNA, such as decreased viability, proliferation, migration, and invasion, as well as increased apoptosis of LUAD cells. *In vivo* studies also demonstrated that A549 cells overexpressing *LOC100270746* induced smaller and fewer lung metastatic nodules in mice, with significantly lower macrophage infiltration in the tumors. Furthermore, *LOC100270746* inhibited M2 macrophage polarization in LUAD. CSF1 is a downstream target of *LOC100270746*, with this lncRNA exerting its effects by suppressing CSF1 expression (Li Y. et al., 2022). In contrast, GNAS-AS1, another lncRNA, showed higher expression in M2 macrophages and NSCLC-related TAMs than in M0 and M1 macrophages. The expression of GNAS-AS1 was significantly higher in tumor tissues from patients with NSCLC than in adjacent normal tissues. Moreover, high levels of GNAS-AS1 were associated with unfavorable clinicopathological characteristics, including lymph node involvement, shorter overall survival, and metastasis-free survival. Inducing the overexpression of GNAS-AS1 in THP-1-differentiated macrophages resulted in greater polarization to M2 macrophages upon stimulation with IL-4, which in turn promoted NSCLC migration and invasion. Additionally, the knockdown of GNAS-AS1 using shRNA significantly reduced the A549 and H1299 cells proliferation, invasion, and migration (Li Z. et al., 2020). *LINC00313* is another lncRNA involved in M2 macrophage polarization. This lncRNA was detected in exosomes from H1299 cells, and its depletion abrogated M2 macrophage polarization. *LINC00313* was found to act as an miR-135a-3p sponge, leading to the upregulation of STAT6 expression (Kong et al., 2022).

In addition to M1 macrophages, exosomes have been found to exert anti-tumor effects, by decreasing the viability, invasiveness, and migration of lung cancer cells (Peng et al., 2023). MiRNAs are one of the functional components of exosomes (Dexheimer and Cochella, 2020) and are involved in various aspects of cancer immunity (Li C. et al., 2022). For example, miR-let-7b-5p was detected at higher levels in the exosomes derived from M1 macrophages compared to M0-derived ones. Investigations have shown that exosomes can transfer miR-let-7b-5p to lung tumor cells, inhibiting tumor cell proliferation and promoting tumor cell apoptosis by regulating GNG5 protein levels (Peng et al., 2023). In another study, miR‐181a‐5p was identified as a key molecule in M1-derived exosomes that inhibits cell proliferation and induces apoptosis by targeting ETS1 and serine/threonine kinase 16 (STK16). STK16 is highly expressed in LUAD tissues, and its expression is associated with an unfavorable prognosis (Wang X. et al., 2022).

Evidence suggests that macrophage polarization plays a role in shaping the response to lung cancer treatment. For instance, anti-silencing factor 1 (Asf1) is a highly conserved chaperone of histones H3/H4. silencing *Asf1a* in *KRAS*-mutant LUAD cells using CRISPR-Cas9 increased tumors sensitivity to anti-PD-1 treatment in orthotopic lung cancer models, without affecting tumor cell proliferation. This enhanced treatment response was attributed to the elevation of M1 macrophages and subsequent T cell activation through granulocyte-macrophage colony-stimulating factor (GM-CSF) upregulation (Li F. et al., 2020). Furthermore, high plasma IL-6 levels in lung cancer patients have been associated with immunotherapy resistance (Kuo et al., 2021). Macrophage-derived IL-6 promotes STAT3-dependent PD-1 expression in CD8^+^ T cells, involving Rab37, a member of the Rab-GTPase family implicated in membrane trafficking. Dysregulation of Rab37 has been observed in lung cancer cells (Tzeng et al., 2017). *Rab37* knockout mice induced with LLC demonstrated the pivotal role of this molecule in PD-1 expression in CD8^+^ T cells, these mice also showed reduced Tregs and CD206^+^ M2 macrophages (Kuo et al., 2021). Therefore, numerous various genes regulate macrophage polarization to either promote or inhibit lung tumor progression.

### 3.5 ILCs, NKT cells, and γδ T cells

Innate lymphoid cells (ILCs) are a subset of the innate immune system that can be activated without antigen-specific receptors in response to alterations in homeostasis and stimulatory signals, particularly at mucosal sites. Previous studies have demonstrated the important role of murine lung ILCs in preserving the integrity of the epithelial barrier and maintaining tissue homeostasis in the lungs following influenza virus infection (Monticelli et al., 2011). Three distinct subsets of ILCs (CD45^+^ Lin^−^ CD127^+^) have been identified in the human lung (De Grove et al., 2016). In the case of NSCLC, a higher frequency of NCR^+^ ILC3s has been reported in tumor tissues compared to adjacent normal lung tissues. In contrast, the frequency of ILC2s is significantly lower. These tumor infiltrating ILC3s express low levels of KIRs, Perforin, and CD94. They also play an important role in the TME by producing proinflammatory cytokines such as IL-22 and TNF and promoting leukocyte recruitment through the secretion of IL-2 and IL-8. The frequency of NCR^+^ ILC3 decreases as the tumor progresses, which is potentially associated with poor prognosis (Carrega et al., 2015). Investigations have also revealed the accumulation of ILCs in the central region of human lung tumors, characterized by lower expression levels of immune checkpoint receptors, such as T cell immunoglobulin and mucin-domain containing-3 (TIM-3), T cell immunoreceptor with Ig and ITIM domains (TIGIT), PD-1, and CD39. Decreased expression of CD49a, in contrast to increased expression of CD103, was observed in ILCs of the tumor center compared with those located in tumor-free distal sites (Brownlie et al., 2023). To understand the specific factors regulating ILC plasticity within the TME of NSCLC, IL-23 producing SqCCs (one of the two types of NSCLCs) were found to induce the conversion of ILC1 to ILC3. IL-17 secreted by ILC3 promotes tumor progression, leading to poor prognosis in patients with SqCCs. This suggests a potential therapeutic target in the IL-23/ILC3/IL-17 axis for IL-23-producing lung tumors (Koh et al., 2019). While several studies have investigated the characteristics of ILCs within the TME of lung cancer and their possible association with prognosis, limited research has explored the immunogenomic landscape of ILCs in various lung tumors. Despite the immunoregulatory functions of ILCs, they may not be ideal candidates for immune checkpoint therapies due to their low expression of immune checkpoint receptors.

Human NK T (NKT) cells, identified as CD3^+^ CD56^+^ or CD8^+^CD56^+^, represent a subpopulation of CD1d-restricted T cells, demonstrating characteristics of both T and NK cells. These cells promote anti-tumor immunity by producing interferon β or participating in the elimination of tumor cells (Yin et al., 2021). NKT1, NKT2, and NKT17 cell subsets have been found in murine lungs, with NKT1 being the most prevalent (Crosby and Kronenberg, 2018). However, the difference in the percentage of NKT cells (CD45^+^CD3^+^CD56^+^) and T cells (CD45^+^CD3^+^CD56^−^) was insignificant between patients with lung cancer and healthy controls (Yin et al., 2021). The immunoregulatory functions of NKT and CD8^+^ T cells in NSCLC are in agreement with the remarkable expression of PD-1 and lymphocyte activation gene 3 (LAG-3) in these cells, as demonstrated by single-cell CyTOF analysis (Datar et al., 2019). CD3^+^CD56^+^CD16^+^ NKT-like cells are potential biomarkers for predicting the response and toxicity of immune checkpoint inhibitors in advanced-stage NSCLC. Further studies with larger cohorts are required to validate these observations (Lin et al., 2023).

**FIGURE 1:**
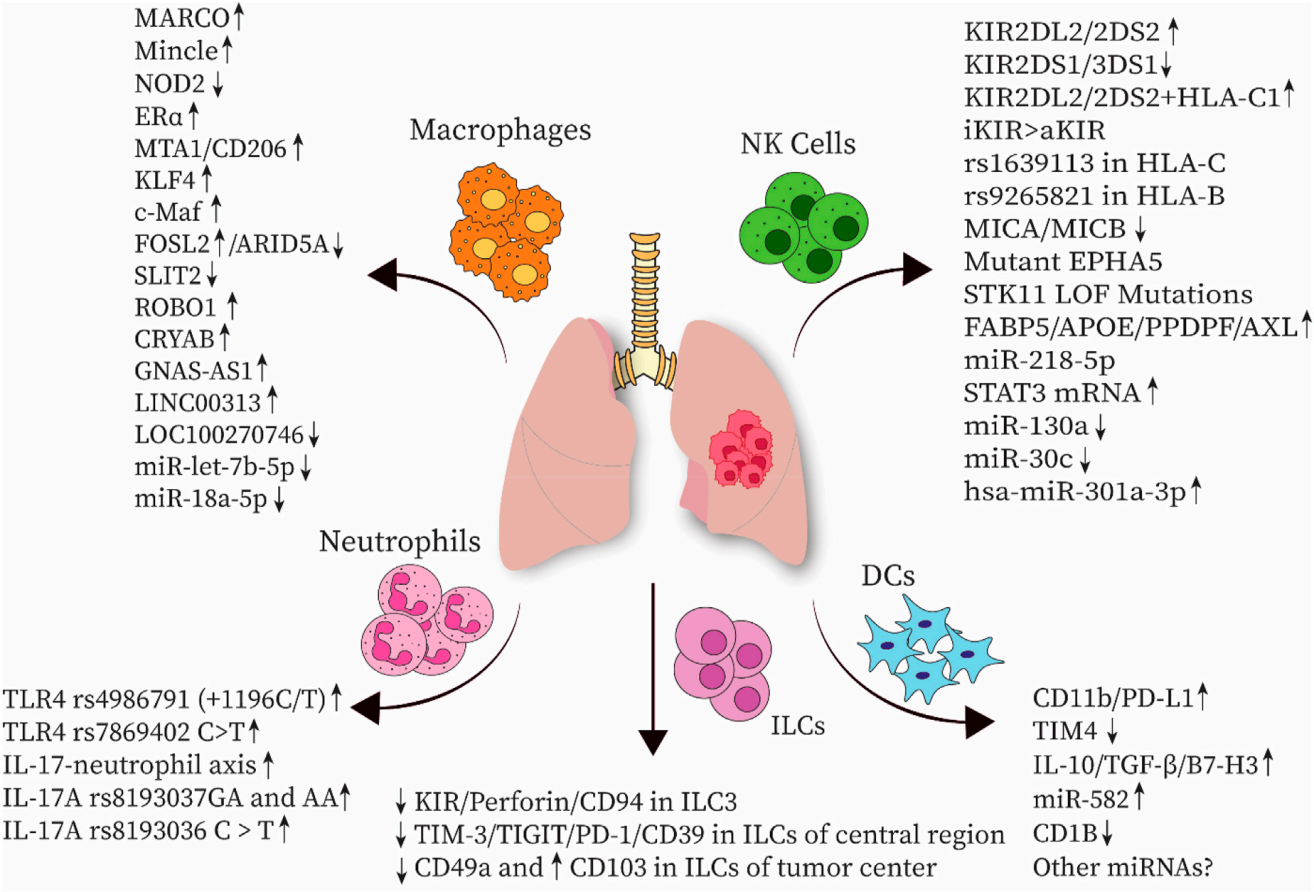
Several immunogenetic factors associated with innate immune cells have been implicated in the progression of lung cancer. NK cell, natural killer cell; DC, dendritic cell; ILC, innate lymphoid cell; MARCO, macrophage receptor with collagenous structure; NOD2, nucleotide-binding oligomerization domain-containing protein 2; Erα, estrogen receptor α; MTA1, metastasis-associated gene 1; KLF4, Krüppel-like factor 4; FOSL2, FOS-like antigen 2; ARID5A, AT-rich interaction domain 5A; SLIT2, slit guidance ligand 2; ROBO1, roundabout guidance receptor 1; CRYAB, αB-crystallin; TLR, Toll-like receptor; KIR, Killer-cell immunoglobulin-like receptor; TIM-3, T-cell immunoglobulin and mucin-domain containing-3; TIGIT, T cell immunoreceptor with Ig and ITIM domains; PD-1, Programmed cell death protein 1; TGF-β, Transforming growth factor-β; TIM4, T-cell immunoglobulin mucin-4; HLA, human leukocyte antigen; MICA, MHC class I polypeptide-related sequence A; EPHA5, erythropoietin-producing hepatocellular receptor A5; STK11, Serine/threonine kinase 11; LOF, loss-of-function mutations; FABP5, Fatty acid-binding protein 5; ApoE, apolipoprotein E; PPDPF, pancreatic progenitor cell differentiation and proliferation factor.

Gamma-delta (γδ) T cells are a subset of T cells that can directly eliminate target cells in an HLA-independent manner, making them efficient players in the front lines of anti-tumor immunity. In contrast, in the context of cancer, a distinctive subset known as Vδ2 Tregs, which are specific types of γδ T cells with regulatory activities and Foxp3 expression, can be induced in the presence of antigen stimulation (IPP/IL-2) and TGF-β1 and IL-15 cytokines *in vitro* (Casetti et al., 2009). γδT17 cells may increase the accumulation and expansion of polymorphonuclear myeloid-derived suppressor cells (PMN-MDSCs), potentially inducing a tumor-suppressive microenvironment that promotes colorectal cancer progression (Wu et al., 2014). Therefore, additional investigations are required to further elucidate the specific roles of γδ T cells in various cancer types. Recent findings indicate that in contrast to most peripheral blood γδ T cells expressing Vδ2, the majority of γδ T cells in lung tissues and tumors express Vδ1. These tissue-resident γδ T cells express higher levels of T cell receptor delta constant (TRDC) compared to other tissue sites, with a higher expression of T cell receptor delta variable 1 (TRDV1) than TRDV2, suggesting that γδ T cells in non-malignant lung tissues are in a steady state. Additionally, CD103 expressing Vδ1 γδ T cells are more frequent in tumors than in non-tumor lung tissues, enhancing their tumor-homing capabilities (Davey et al., 2017; Wu et al., 2022). Based on the expression of CD45RA and CD27, γδ T cells can be divided into two groups: effector and memory. Vδ1 T cells with a CD45RA^−^CD27^−^ effector memory T cell (TEM) phenotype are more abundant in tumor tissues than in non-tumor tissues, indicating cytolytic and IFN‐γ production capabilities. Vδ1 T cells show potential for NSCLC immunotherapy; however, they have limited responsiveness to activation-induced cell death, which restricts their suitability for adoptive cell therapy (Davey et al., 2017; Wu et al., 2022). Studies have also shown that DNA methyltransferase inhibitors can enhance the antitumor effects of MHC-unrestricted γδ T cell therapy by increasing the expression of adhesion molecules such as intercellular adhesion molecule 1 (ICAM-1) or facilitating immune cytoskeleton reorganization and immune synapse formation, finally improving the tumor-killing efficacy of γδ T cells. Therefore, epigenetic modifications could be used to develop novel therapeutic strategies for lung cancer and promote cell-based immunotherapy (Weng et al., 2021). Allogeneic Vγ9Vδ2 T cells, which are the most frequent subset of γδ T cells in humans, have been implicated in adoptive immunotherapy. However, further clinical investigations are needed in this field (Xu et al., 2021). Figure 1 shows the recent immunogenetic factors associated with lung cancer progression in innate immunity.

## 4 Cytokines and chemokines

Crosstalk between tumor cells and tumor-infiltrating lymphocytes (TILs) in the TME is mediated by low-molecular-weight secreted proteins, such as cytokines and chemokines. Given their substantial role in creating an inflammatory microenvironment, these TIL-producing proteins can significantly enhance tumor progression, metastasis, and resistance to therapy, thereby emerging as cancer prognostic markers. IL-6, IL-10, IL-2, IL-22, IL-8, IL-32, IL-37, and TNF are the most common cytokines involved in the therapeutic management of lung cancer (Sarode et al., 2020b). Interest in investigating genetic alterations within cytokine and chemokine genes is growing, as these changes can be associated with tumor progression or suppression and may serve as potential prognostic markers. In this regard, the overexpression of tumor necrosis factor superfamily 15 (TNFSF15), a member of TNF superfamily, is linked to suppressed tumor progression in different cancers, primarily through the inhibition of angiogenesis (Hou et al., 2005; Liang et al., 2011). A case-control study on Han Chinese population demonstrated that *TNFSF15*–638A > G and −358 T > C polymorphisms probably increased susceptibility to SCLC, as opposed to NSCLC (Gao et al., 2019). These polymorphisms affect the expression levels of TNFSF15 and its downstream signaling pathways, ultimately modulating the immune response. For instance, the interaction between death receptor 3 (DR3) and vascular endothelial growth inhibitor (VEGI)-251 splice variant, encoded by *TNFSF15*, stimulates signaling pathways by inducing NF-kB and Caspase cascade, initiating T cell responses and cell apoptosis, respectively (Migone et al., 2002; Bittner et al., 2016). Additionally, the *TNFSF15*−358 T > C polymorphism increases the risk of SCLC among nonsmokers rather than smokers, indicating the need for further analysis. Moreover, the *TNFSF15*–638GG genotype is associated with an increased risk of SCLC in men and individuals over 60 years of age (Gao et al., 2019). Higher mRNA and protein expression of TNFα‐induced protein 2 (*TNFAIP2*), a gene regulated by TNF, has been reported in NSCLC tissues compared to adjacent normal tissues, which is likely epigenetically controlled by miR-145-5p. TNFAIP2 plays a role in promoting tumor proliferation, migration, and metastasis in NSCLC. Therefore, interventions such as silencing *TNFAIP2* or overexpressing miR-145-5p may be potential therapeutic approaches for NSCLC (Li J. et al., 2020).

Upregulation of IL-32, a pro-inflammatory cytokine produced by NK cells and functional T cells, has been reported in most LUADs as well as in some large-cell carcinomas and SCLCs, but not in SCCs. TILs and tumor cells expressing IL-32 are associated with lymph node metastasis and poor outcomes (Sorrentino and Di Carlo, 2009). Wang et al. demonstrated an association between the CC homozygote of the rs12934561 *IL-32* polymorphism and a higher risk of lung cancer, whereas the TT genotype was associated with poor survival in patients with squamous carcinoma. The T allele of the rs28372698 *IL-32* polymorphism is associated with poor prognosis in patients with moderately and well-differentiated lung cancer. Moreover, the expression of IL-32 was significantly inhibited in peripheral blood leukocytes of patients with lung cancer (Wang et al., 2017). Conversely, IL-32γ may suppress lung tumor progression by increasing tissue inhibitor of metalloproteinase 3 (*TIMP-3*) expression, which acts as a tumor suppressor gene (Yun et al., 2018), or by downregulating integrin alpha V (ITGAV)-mediated STAT5 signaling pathways, leading to the suppression of the growth of CD133^+^ lung cancer stem cells (Lee et al., 2019b). A recent study conducted in a Brazilian Amazon population has revealed an association between the Ins/Ins genotype of *IL-1A* (rs3783553) polymorphism and a higher risk of NSCLC (Pereira et al., 2023). In hepatocellular carcinoma, this particular polymorphism has been found to increase the expression of IL-1A, potentially by impairing the binding sites of miRNA-122 and 378 to the 3’ UTR region (Gao et al., 2009). A similar mechanism may occur in other cancer types. Given that IL-1A is a pro-inflammatory cytokine mainly secreted by monocytes and macrophages and plays crucial roles in angiogenesis, proliferation, and tumor migration, its upregulation is associated with a higher risk of cancer (Ma and Zhou, 2016). Similarly, IL-1B, mostly secreted from tumor-associated macrophages as a pro-inflammatory cytokine, enhances the expression of ELF3 in LUADs with *EGFR* mutations, leading to the activation of the PI3K/Akt/NF-κB pathway and consequently contributes to tumor progression (He et al., 2021). A case-control study conducted on a Spanish population revealed an association between the *IL1B* rs1143634-TT genotype and a reduced risk of NSCLC, whereas individuals carrying the C allele had an increased risk of developing NSCLC. Several factors, such as the inclusion of different types of lung cancer besides NSCLC, different age groups, and populations from diverse ethnic backgrounds, contributed to the variations observed in the outcomes of this study when compared to previous research. Therefore, further investigations with larger cohorts from different populations are necessary to validate these findings (Pérez-Ramírez et al., 2017). Additionally, high expression of IL-35 was found in the BALF from the tumor site in patients with NSCLC. IL-35 inhibits the function of Th1 and Th17 cells while increasing the regulatory function of CD4^+^ T cells and reducing the cytolytic activity of CD8^+^ T cells, highlighting its role in the induction of T cell exhaustion and dysfunction, favoring tumor progression (Wang et al., 2018). Studies on the Han population have suggested an association between an increased frequency of monocyte chemoattractant protein 1 (*MCP-1*) AA genotype and a decreased frequency of the GG genotype with a higher risk of NSCLC. While they indicateds a role for *MCP-1* polymorphisms in the risk of NSCLC, no association was found between *CCR2* polymorphisms and NSCLC (Yang et al., 2015). However, a significant association was observed between carrying the −64I allele of *CCR2-64I* gene polymorphism and higher susceptibility to NSCLC in a Tunisian population, with a higher expression of CCR2 in patients (Rafrafi et al., 2015). An *in silico* study also indicated lower expression of CCR2 in patients with LUAD, while higher expression was associated with better survival in these patients (Wan et al., 2021). Given these controversies, further research in larger samples is required to clarify the role of *CCR2* polymorphisms in lung cancer.

A GWAS conducted in a Croatian population consisting of 203 patients with lung cancer and 360, 938 controls demonstrated a significant association between specific SNPs and increased susceptibility to lung cancer. These SNPs include rs6806802 in *CCR9*, rs9766026 in *F13A1*, rs2386841 in *IL2RA*, rs2495366 in *TNRSF14*, rs1148471 in *TNRSF8*, rs1682802 in *GNA15*, and rs1148038 in *IL31RA*. These genes and associated SNPs were mainly involved in the activation of NF-κB transcription factor, leading to an inflammatory condition. Given that the higher number of controls compared with cases in this GWAS may increase the possibility of false-positive/negative findings; therefore, further investigation is required to confirm these findings (Baranasic et al., 2023). Table 1 summarizes the other cytokine polymorphisms associated with lung cancer susceptibility and protection.

**TABLE 1:** Recent cytokine polymorphisms associated with lung cancer development or suppression.

Gene	Population	Ethnicity	Method	Polymorphisms associated with higher or lower risk of lung cancer	References
*IL1B*	627 cases and 633 controls	Northeastern-Chinese	LDR-PCR	-Protective effect of variant G-allele of rs1143633 in smoking subgroup of >20 years -Association of *IL1B* SNP rs1143633 with lower risk of lung cancer -Association of *IL1B* SNP rs3136558 and haplotype4 consisting of *IL1B* htSNPs (rs1143633A-rs3136558C-rs1143630A) with increased risk of lung cancer	Yin et al. (2023)
*IL-8*	358 cases and 716 controls	Taiwanese	PCR-RFLP	-Lower percentage of the homozygous variant AA *IL-8* rs4073 genotypes in the case group compared to the control group -Association of *IL-8* rs4073 genotypes with lung cancer susceptibility, particularly for smokers	Li et al. (2022c)
*IL‐1R2*	259 cases and 346 controls	Han Chinese	Agena MassARRAY RS1000 system	-Association of rs3218977‐GG with a decreased risk of lung cancer -Significant risk-increasing effect of rs2072472 in the dominant model -rs2072472 as the most influential risk factor of lung cancer -Downregulation of *IL‐1R2* mRNA level in lung cancer patients -Association between high expression of IL‐1R2 and a poor prognosis in lung cancer	Wang et al. (2019)
*IL-6* *IL-8* *IL-17A/F* *MIF*	160 cases and 150 controls	Moroccan	PCR-RFLP	-A significant association between *IL-6* (rs1800795, rs1800796), *IL-8* (rs4075, rs2227306), *IL-17F* (rs763780, rs2397084) and *MIF* (rs755622) and the risk of lung cancer -A significant difference in mRNA expression levels of *IL-6*, *IL-8*, *IL-10*, *IL-17* and *TNF* genes in lung cancer patients compared to healthy subjects	Kaanane et al. (2022)
*IL-12A/B*	358 cases and 716 controls	Taiwanese	PCR-RFLP	-Association of AA genotype of *IL-12A* rs568408 with a significantly elevated risk of lung cancer compared with the GG genotype, especially among those with smoking habits	Wu et al. (2018)
*IL-31*	302 cases and 493 controls	Chinese	TaqMan probe real-time PCR	-Association of *IL-31* polymorphisms with susceptibility and survival status in lung cancer -Suggesting a role for IL-31 to be used as a biomarker along with other tumor-associated markers -Higher probability to lung cancer development in patients with CG genotype of rs7977932 or CA genotype of rs4758680 -An increased risk of metastasis and lower survival rate in patients with rs7977932 CC genotype	Yang et al. (2018)

LDR-PCR: ligase detection reaction coupled with polymerase chain reaction; htSNP: haplotype-tagging single nucleotide polymorphism; PCR-RFLP: polymerase chain reaction-restriction fragment length polymorphism; MIF: macrophage migration inhibitory factor; TNF: tumor necrosis factor.

Various miRNAs have been shown to regulate the expression of cytokine or chemokine genes, influencing tumor progression and ultimately patient survival. The expression of miR-3648 was significantly elevated in LUAD cell lines (PC9 and A549) and LUAD tissues compared to normal lung epithelial cells (BeAS-2B) and noncancerous tissues, respectively. miR-3648 promotes tumor cell progression by suppressing the expression of cytokine signaling 2 (SOCS2), a member of the SOCS family that negatively regulates cytokine receptor signaling. Therefore, miR-3648 could be considered a potential target for LUAD immunotherapy (Tu and Mei, 2022). Similarly, miR-875 was overexpressed in NSCLC tumor tissues compared to normal adjacent tissues. It regulates tumor proliferation and apoptosis by targeting SOCS2, indicating its potential as a therapeutic target (Tong et al., 2019). Studies have also shown higher expression of miR-20a and lower expression of miR-145 in NSCLC serum compared to controls, suggesting them as potential biomarkers. Furthermore, a weak to moderate association between the concentrations of TGF-β and VEGF, and miR-20a and miR-223 has been reported, indicating them as potential targets for lung cancer treatment (Chaniad et al., 2020). In patients with SCC, miR-9-3p negatively regulates the expression of IL-17A. IL-17 is a cytokine with both antitumor and protumor effects; in the case of SCC, miR-9-3p modulates the expression of IL-17A as an antagonist of IL-17A mRNA, which is crucial in SCC development (Migdalska-Sęk et al., 2020). Lower expression of miR-195-5p has been reported in NSCLC tissues and cell lines and is associated with poor survival in patients with NSCLC, suggesting its role as a prognostic marker (Zheng et al., 2019). Previous studies have shown an association between miR-200 family members and decreased overall survival in NSCLC patients, particularly in those with LUAD. Further investigation revealed that miR-200a-3p increased the expression of CX3CR1, leading to the induction of epithelial-to-mesenchymal transition (EMT) and distant metastasis. This finding suggests that the miR-chemokine receptor axis could be considered a therapeutic target in patients with LUAD. The expression of CX3CR1 and CXCR1 chemokine receptors is correlated with the infiltration of M2 macrophages and NKT cells, indicating their potential role in regulating immune responses in the TME of LUAD (Sharma et al., 2023). TGF-β contributes to tumor progression by inducing EMT. *LINC00273*, a long noncoding RNA, liberates Zinc finger E‐box binding homeobox 1 (ZEB1) from miR‐200a‐3p, promoting TGF-β-induced lung tumor metastasis, suggesting *LINC00273* as a target for preventing lung tumor metastasis (Sarkar et al., 2020). IL-13 has been implicated in promoting lung cancer progression by increasing the expression of the Ying Yang 1 (YY1) transcription factor through the activation of the PI3K/AKT pathway in A549 cells, leading to enhanced migration and proliferation of these cells. The pro-tumorigenic effects of IL-13 are inhibited by miR-29a by targeting YY1. However, further *in vitro* and *in vivo* studies are required to validate and better understand the therapeutic potential of miR-29a in lung cancer (Zhang et al., 2018).

Environmental factors play a significant role in modulating cytokine secretion, thus supporting the progression of cancer. For instance, prolonged exposure to particulate matter 2.5 (PM2.5), a crucial component of air pollution, results in the upregulation of transmembrane serine protease 2 (TMPRSS2) in lung cancer cells. This, in turn, fascilitates inflammasome formation and increases the secretion of IL-18, a cytokine associated with increased cell proliferation, pro-inflammatory responses, and cancer development (Wang et al., 2023).

In conclusion, the type of lung cancer, the ethnicity of the study population, and the sample size may have contributed to the diverse findings related to cytokine polymorphisms. However, analyzing high-throughput data using GWAS can result in more reliable outcomes, enabling the identification of polymorphisms that can be used as prognostic markers or potential targets for lung cancer treatment. Despite the need for further analysis to better understand the association between different miRNAs and cytokine or chemokine expression, more efforts should be directed towards identifying specific sets of miRNAs that can be used as predictive indicators or potential targets in lung cancer therapy.

## 5 Adaptive immunity

### 5.1 B cells

In a study by Li et al., a gene signature consisting of pairs of genes related to B cells was constructed by analyzing scRNA-seq data from patients with NSCLC. This gene signature has proven to be a strong prognostic factor for patients with NSCLC, showing a significant correlation with immune scores, tumor purity, clinicopathological characteristics, and various tumor-infiltrating immune cells. Furthermore, this signature has shown promising results as a potential biomarker for predicting the efficacy of immunotherapy in patients with NSCLC (Li X. et al., 2022). Moreover, the samples of 11 patients with NSCLC were categorized into B‐cell rich and B‐cell poor groups using RNA-seq data. B‐cell rich samples exhibited elevated levels of the CXCL13 transcripts, a recognized B‐cell chemoattractant, and PD1^+^ CD8 T cells (Raju Paul et al., 2023). CXCL13 and PD-1^+^ CD8 T cells play key roles in the formation of tertiary lymphoid structures, which have been identified in several cancers and are typically associated with favorable outcomes (Sautès-Fridman et al., 2019; Workel et al., 2019). B cells, which contribute to and regulate these structures, were significantly correlated with tertiary lymphoid structures in NSCLC samples. Moreover, RNA-seq data of patients with LUAD who underwent surgery, chemotherapy, or radiation revealed that pre-treatment percentage of intratumoral B cells correlated with improved overall survival in all stages of LUAD (Raju Paul et al., 2023). Transcriptomic analysis has also revealed that the presence of B and plasma cells in the TME plays a crucial role in determining the overall survival of patients with NSCLC treated with atezolizumab. B cells and plasma cells are also associated with the presence of tertiary lymphoid structures (Patil et al., 2022). Another study using RNA-seq data extracted from the TCGA database showed that patients with high B cell infiltration in LUAD had longer survival compared to those with low infiltration. However, the presence of PD-L1 may impede the survival advantage among patients with high B cell infiltration (Ho et al., 2018). Future research could focus on exploring the association between B cell receptor (BCR) genetics and lung cancer, as no investigations have been conducted in this area so far.

### 5.2 T cells

CD4^+^ T cells are the most common T cell population in adenocarcinoma and SCC, followed by CD8^+^ cells. A small portion of TILs consists of double negative T cells, with the CD45RA^−^CD45RO^+^ memory/effector phenotype being the most prevalent among T cells (Stankovic et al., 2018). Genetic alterations can significantly modify T cell immune responses, affecting the clinical outcomes of lung cancer patients. Effective immune responses to TILs depend on T cell receptor (TCR)-mediated recognition of mutation-derived neoantigens. Therefore, exploring the TCR repertoire has the potential to predict responses to immune checkpoint blockade and survival in cancer patients. A cold and heterogeneous TCR repertoire is associated with poor prognosis in patients with localized NSCLC. To investigate this correlation, a study conducted TCR sequencing of 45 tumor regions from 11 patients with localized NSCLC and discovered extensive intratumor heterogeneity (ITH) in the TCR repertoire in terms of T cell density and clonality, which are factors related to T cell response and expansion. The observed spatial differences in the TCR repertoire were attributed to the distinct neoantigens in different tumor regions (Reuben et al., 2017; Reuben et al., 2020). High molecular genomic ITH, defined as the presence of various cancer cells and stromal cells with diverse molecular and phenotypic characteristics within the same tumor, has been associated with a higher risk of relapse, lower response to treatment, and increased tumor aggressiveness (Zhang et al., 2014; McGranahan et al., 2016; Stewart et al., 2020). Higher ITH in T cell clonality has also been linked to an increased risk of postsurgical relapse. These mutations, occurring in different regions of a tumor, can result in the differential expression of neoantigens, leading to variations in immunogenicity and the ability of different tumor regions to induce an anti-tumor T cell response. Previous studies have indicated that tumors with high clonal neoantigen burden and low neoantigen ITH are associated with a significantly longer progression-free survival (McGranahan et al., 2016). These findings emphasize the significance of taking into account the quantity and heterogeneity of neoantigens when predicting the prognosis and clinical outcomes of patients with lung cancer. T cell heterogeneity may result from heterogeneity in the architecture of tumors, as well as their vasculature and lymphatics, which in turn can affect the ability of T cells to infiltrate different regions of a tumor. Therefore, analyzing the textural features of patients’ tumors, possibly through noninvasive methods, to detect ITH in the mutational landscape or T cell repertoire could provide valuable insights for predicting how patients will respond to therapies (Reuben et al., 2017). Higher TMB has also been associated with increased T cell clonality, a lower CD4:CD8 ratio, higher levels of granzyme B, higher immunogenicity, and the induction of T cell responses through the creation of neoantigens. Moreover, higher homology in the T cell repertoire between the tumor and uninvolved tumor-adjacent lung in patients with NSCLC is linked to lower survival, likely due to the decreased tumor-focused T cell responses. When performing T cell expansion for therapeutic purposes, it is crucial to consider the overlap of potentially reactive T cells between uninvolved tumor-adjacent lungs and tumor tissues in patients with NSCLC. This approach is essential to avoid non-specific anti-tumor responses and minimize the risk of immune-related adverse events associated with the expansion of T cell subsets unrelated to tumors. It should also be taken into account when developing and implementing TIL-based therapies (Reuben et al., 2020). In case of SCLC, whole-exome sequencing of the TCR was conducted on 50 tumor samples from 19 resected limited-stage SCLC tumors (LS-SCLCs). The results revealed a high variation in TMB and copy number aberration (CNA) burden between patients, while it was similar between different regions within the same tumor. SCLC tumors also demonstrate lower TCR metrics, including diversity, density, and clonality, than tumor-adjacent lung tissues, indicating reduced infiltration, proliferation, and diversity of T cells in tumor tissues. A high CNA burden is negatively associated with the quality and quantity of T cells, probably contributing to a cold TCR repertoire in SCLC and the cold TME. Collectively, higher levels of TCR ITH have been identified in SCLC tumors than in NSCLC, potentially compromising the efficacy of anti-tumor immune responses (Chen et al., 2021). Additionally, copy-number loss of IFN-γ pathway genes has been reported in SCLC samples, positively associated with CNA burden This suggests a potential mechanism underlying immune evasion in SCLC tumors. This phenomenon may be attributed to dual inactivation of TP53 and RB1, chromosome instability, and ultimately the loss of essential immune related genes such as IFN-γ pathway genes This allows SCLC tumors to evade the anti-tumor immune system. Similar to NSCLC, a higher TMB was associated with longer overall survival, while a higher CNA burden was linked to lower overall survival. Furthermore, patients with a more heterogeneous TCR showed a significantly shorter overall survival. In summary, a cold and heterogeneous TCR repertoire may be one of the factors leading to a lower response to immunotherapy in patients with SCLC. Therefore, overcoming the cold and heterogeneous intratumor T cell repertoire may help enhance the efficacy of immunotherapies in patients with SCLC (Chen et al., 2021).

Further research has also been conducted to clarify the correlation between immune system-related genes and T cell response against lung cancer. A study of 2450 SNPs in T cell cancer immune response-related genes in 941 patients with early-stage NSCLC revealed that rs1964986 and rs1573618 SNPs in the TCR beta chain (*TRB*) were associated with early-stage NSCLC recurrence. The rs10108662 SNP in indoleamine-2,3-dioxygenase 1 (*IDO1*) was associated with a higher risk of death, potentially affecting NSCLC patient outcomes by modulating the expression of IDO and T cell cytotoxicity. Additionally, rs959260 and rs4789182 SNPs in the growth factor receptor-bound protein 2 (*GRB2*) gene were associated with survival, likely due to their role in regulating immune cell development and T cell functions. The rs8080546 SNP in the proteasome 26S subunit, non-ATPase 3 (*PSMD3*), was associated with a higher risk of death in patients who only underwent surgery, possibly because of increased MHC-1 peptide presentation, leading to T cell exhaustion and impaired T cell cytotoxicity (Wang Q. et al., 2020).

RAS guanyl-releasing protein 2 (RASGRP2), one of the guanine nucleotide exchange factors (GEFs), has been implicated in chemokine and antigen-receptor/cytokine-cytokine receptor signaling pathways, T cell activation, and lymphocyte differentiation in LUAD. RASGRP2 expression has been lower in LUAD tissues than in non-tumoral tissues, and lower levels of RASGRP2 correlated with a poor prognosis Studies have also indicated a positive association between the expression of RASGRP2 and the infiltration of immune cells, including cytotoxic T cells, Th1 cells, and B cells, and a negative association with the infiltration of Th2 cells. Positive correlations have also been reported between RASGRP2 expression and certain immunoinhibitors (such as CTLA-4), immunostimulators (such as TNFRSF13B), chemokines (such as CCL14/17/19), and chemokine receptors (such as CCR6/7 and CXCR5). Immunofluorescence experiments have suggested a potential association between higher levels of RASGRP2 and PD-L1 expression. This implyies that patients with increased RASGRP2 expression may benefit more from immunotherapy. The effects of RASGRP2 may occur through the JAK3-STAT5 signaling pathway, although comprehensive animal experiments and clinical trials are necessary for confirmation (Liu et al., 2023). A recent study found that Foxp3 expression is higher in NSCLC tissues compared to normal tissues. Immunohistochemical (IHC) analysis showed that Foxp3 is mainly expressed by Tregs rather than in cancer tissues. The increased expression of Foxp3 is associated with worse overall survival, indicating that Tregs could be a possible target for therapy in patients with NSCLC (Zhu et al., 2022).

Moreover, polymorphisms observed at several T cell immune checkpoints, such as B- and T-lymphocyte attenuator (*BTLA*), *CTLA-4*, and *PD-1*, which are known for their immunosuppressive roles, have been implicated in modulating susceptibility to lung cancer (Wang J. et al., 2021; Andrzejczak et al., 2022). Table 2 illustrates the identified polymorphisms in T cell immune checkpoints and their association with an increased or decreased risk of lung cancer.

**TABLE 2:** Identified polymorphisms in immune checkpoints and FOXP3 associated with lung cancer development or suppression.

Gene	Population	Ethnicity	Method	Polymorphisms associated with higher or lower risk of lung cancer	References
*BTLA*	383 NSCLC patients and 474 or 309 controls (depending on SNPs)	Caucasian	Real-Time PCR using TaqMan probes	-Association of the rs1982809 within *BTLA* with higher risk of NSCLC -Increased risk of NSCLC in females having the rs1982809G (AG + GG genotypes) allele and the rs9288953T allele (CT + TT genotypes) -Correlation of the rs1982809G and rs2705511C with the more advanced stages of NSCLC (stage II and III) -Significant overrepresentation of rs1982809G carriers in never-smokers	Andrzejczak et al. (2022)
*BTLA*	1,003 NSCLC patients and 901 controls	Chinese	SNPscan™ Kit (PCR)	-Association of the *BTLA* rs1982809 polymorphism with reduced risk of NSCLC -Association of the *BTLA* rs16859629 polymorphism with increased risk of squamous cell carcinoma	Wang et al. (2021c)
*BTLA*	169 lung cancer patients and 300 controls	Tunisian	TaqMan SNP Genotyping Assay	-Association of the rs1982809 SNP with an increased risk of lung cancer compared with controls in codominant and dominant models -Higher risk of developing lung cancer in the heterozygous rs1982809-AG genotype carriers when compared to AA genotype carriers -The AG genotype as an important risk factor and its association with lymphatic invasion and large-sized lung tumor	Khadhraoui et al. (2020)
*TIM-3*	432 NSCLC cases and 466 controls	Han Chinese	PCR-RFLP	-About 2.81-fold increased risk of NSCLC in subjects carrying the +4259TG genotype -Shorter survival time in cases with +4259TG genotype	Bai et al. (2013)
*FOXP3*	192 NSCLC patients and 259 controls	Han Chinese	PCR-RFLP	-Association between the A allele of rs3761548 and increased risk of NSCLC -Association of the AC genotype, AA genotype, and the combined A variant genotype (AA + AC) with a higher risk of NSCLC -A significantly higher frequency of AA + AC genotype in patients with stage II NSCLC	He et al. (2013)
*PD-1*	287 patients with LUAD and 111 controls	Han population of Northeast China	PCR and SNaPshot Multiplex Kit	-The rs2227981, rs2227982, rs3608432, and rs7421861 SNPs as potential markers to distinguish between early and late stages of LUAD	Huang et al. (2019)
*PD-1* *TIM-3*	383 NSCLC patients (112 with LUAD and 116 with LUSC) and 433 controls	Polish	PCR–RFLP and TaqMan SNP Genotyping Assays	-Two times higher risk of death in NSCLC carriers of rs11568821 T allele compared to carriers of CC genotype -The rs10057302 CA genotype as an independent predictor of overall survival in LUSC	Moksud et al. (2023)
*CTLA-4*	71 patients with NSCLC and 104 controls	Polish	Real-Time PCR System using TaqMan 5′allelic discrimination assay	-Increased CTLA-4 expression in the majority of NSCLC patients -Association of the presence of G allele and GG genotype in cancer tissue (+49A/G) with the increased NSCLC risk	Antczak et al. (2013)
*CD28*, *ICOS*, and *CTLA-4*	208 NSCLC patients and 326 controls	Caucasian	PCR-RFLP	-Increased risk of NSCLC about 2-fold with the constellation of alleles *CTLA*-4c.49A>G [A]/CT60 [G]/*CD28*c.17 + 3T>C [T]/*ICOS*c.1554+4GT (8_15)[>10]	Karabon et al. (2011)
*CTLA-4*	338 patients with advanced NSCLC	Chinese	PCR-RFLP	-Significantly shorter survival time in patients with the AA genotype compared to those with the GG genotype or the GA genotype -The polymorphism of *CTLA-4* +49A1G as a prognostic predictor for advanced NSCLC	Song et al. (2011)
*CTLA-4*	127 lung cancer patients and 124 healthy controls	Iranian	PCR-RFLP	-No significant association between six main SNPs of the *CTLA-4* gene (−1722T/C, −1661 A/G, −318 C/T, +49A/G, +1822 C/T, and +6230 A/G (CT60)) and susceptibility to lung cancer	Khaghanzadeh et al. (2010)

BTLA: B- and T-lymphocyte attenuator; NSCLC: Non-small cell lung cancer; CTLA-4: Cytotoxic T lymphocyte antigen 4; PCR-RFLP: Polymerase Chain Reaction-Restriction Fragment Length Polymorphism; SNP: Single Nucleotide Polymorphism; TIM-3: T cell immunoglobulin and mucin domain-containing protein 3; FOXP3: Forkhead Box Protein P3; PD-1: Programmed Cell Death Protein 1; ICOS: Inducible T Cell Co-stimulator; LUAD: lung adenocarcinoma; LUSC: squamous cell lung cancer.

*PD-L1* polymorphisms have also been associated with the susceptibility to lung cancer. A study of 288 patients with NSCLC and 300 controls found a higher frequency of the *PD-L1* 8923A/C polymorphism in NSCLC patients compared to controls. These patients also had elevated levels of soluble PD-L1, which were higher in LUAD patients than in those with SCC (Cheng et al., 2015b). Moreover, the CC genotype of rs4143815 and the GG genotype of rs4742098 SNPs in *PD-L1* were associated with a nearly two-fold higher risk of squamous cell lung cancer (LUSC). This polymorphism likely affects the interaction between hsa-mir-570 and CD274 mRNA (Wang et al., 2013; Moksud et al., 2023). Another study of *PD-L1* polymorphisms was conducted on 126 Egyptian patients with lung carcinoma and 117 healthy controls. The results showed an association between rs2297136 and rs4143815 variants with a higher and lower risk of lung cancer, respectively, suggesting they could be potential biomarkers for lung cancer (Sakran et al., 2023).

## 6 Immunogenetics and response to immunotherapies

Nivolumab, an FDA-approved IgG4 anti-PD-1 monoclonal antibody for NSCLC, is used after platinum-based chemotherapy for patients with high or low PD-L1 expression. Pembrolizumab, another IgG4 anti-PD-1 monoclonal antibody, is used to treat metastatic NSCLC with over 50% PD-L1 expression, but without *EGFR* or *ALK* mutations. If PD-L1 expression is less than 50%, it should be combined with other treatments. Atezolizumab, an IgG1 antibody against PD-L1, is suitable for patients with metastatic NSCLC expressing *EGFR* or *ALK* mutations, administered during or after platinum-based chemotherapy (Lindeman et al., 2013; Ramos-Esquivel et al., 2017; Siddiqui and Siddiqui, 2023). Nivolumab (IgG4 anti-PD-1) in combination with ipilimumab (fully humanized IgG1 anti-CTLA4) and minimal chemotherapy (only two cycles of platinum-based) has shown efficacy comparable to full-dose chemotherapy in patients with metastatic NSCLC (Shalata et al., 2023). Immunotherapies using anti-PD-1, anti-PD-L1, and anti-CTLA-4 monoclonal antibodies have revolutionized lung cancer treatment; however, only a few advanced-stage patients respond. The high degree of heterogeneity among tumor types and genomic alterations makes it difficult to predict which patients will benefit from immunotherapy (Wang et al., 2022b). Several factors, including mismatch repair deficiency (dMMR)/microsatellite instability (MSI), TMB, tumor PD-L1 expression, and host-specific immunity characteristics, such as the microbiome or germline genetics of the host, can affect the response to immune checkpoint inhibitor-based therapy by determining the immunogenicity of tumors (Le et al., 2015; Ott et al., 2019; Baruch et al., 2021; Doroshow et al., 2021; Sayaman et al., 2021). Additionally, treatment with anti-PD1 blocking antibodies may result in toxicities, including dermatitis, hypothyroidism, colitis, and pneumonitis. Studies aimed at identifying patients more susceptible to such adverse effects could help in selecting personalized therapies. In this regard, Bins et al. investigated whether patients with SNPs in *PD-1* and *PD-L1*-related genes experience severe toxicity with anti-PD1 blocking antibodies. They concluded that these SNPs are unlikely to have clinical implications in predicting nivolumab toxicity in patients with NSCLC (Bins et al., 2018).

Several studies have investigated the mechanisms underlying resistance to immune checkpoint inhibitors. For example, the *KIR3DS1* genetic variant of *KIR* is significantly associated with resistance to PD-1 blockade and progression-free survival in patients with NSCLC, suggesting a potential role for NK cells in responding to PD-1 immunotherapy (Trefny et al., 2019). Lower serum levels of granzyme B and homozygous and heterozygous variants of *GZMB* rs8192917 are associated with poor prognosis and worse clinical outcomes with PD-1 blockade in patients with NSCLC, highlighting the importance of considering T cell response-related mutations in predicting NSCLC prognosis and response to immunotherapy (Hurkmans et al., 2020). TMB, which is associated with the number of tumor-derived neoantigens, is a significant determinant of a favorable response to immune checkpoint inhibitors. These neoantigens are subsequently presented by MHC molecules, activating T cell anti-tumor responses (McGranahan et al., 2016; Samstein et al., 2019). In addition to TMB, heterozygosity in the repertoire of patient-specific antigen-presenting HLA-I molecules also affects the response to immune checkpoint blockade (ICB) by influencing the selection and clonal expansion of T cells reactive against tumor-derived neoantigens after ICB treatment. Heterozygous *HLA-I* genotypes facilitate the presentation of a diverse range of tumor-derived antigens to T cells. In this regard, homozygosity at one *HLA-I* locus (A, B, or C) and low TMB were associated with a significant reduction in overall survival compared to patients with heterozygosity at each *HLA-I* locus and high TMB. These factors should be considered in future clinical trials (Chowell et al., 2018). Recent studies have also demonstrated that patients with high HLA-I evolutionary divergence (HED), as measured by the sequence divergence between alleles of the *HLA-I* genotype, are likely to be more responsive to ICB therapies. This factor can be determined by DNA sequencing of normal tissues, as opposed to TMB, which is challenging to evaluate because of tumor purity and clonal fraction. All of these factors, such as TMB, HED, and TCR repertoire, can predict T cell-mediated tumor control and should be considered in future clinical investigations (Chowell et al., 2019). Considering different aspects of the interaction between the tumor and TME is important for predicting the response to immunotherapy. A study involving different tumor regions from 88 patients with early-stage untreated NSCLC demonstrated that the immune system of these patients imposes strong selection pressure during tumor evolution, contributing to immune evasion and poor disease-free survival (Rosenthal et al., 2019). Immunoediting mechanisms may occur by decreasing the expression of neoantigens and HLA LOH, enabling cancer cells to escape immune recognition by deleting HLA alleles, thereby suppressing neoantigen presentation (Pyke et al., 2022) or promoter hypermethylation of genes encoding neoantigens. Therapeutic interventions should aim to mitigate or inhibit immune evasion mechanisms (Rosenthal et al., 2019).

Studies have suggested that patients with NSCLC lacking driver oncogenes may demonstrate a more favorable response to immune checkpoint inhibitor therapy than patients with oncogene-addicted NSCLC, particularly those with *EGFR/ALK* variations (Lisberg et al., 2018; Addeo et al., 2021). However, whether the immunogenicity and tumor immune microenvironment (TIME) of HER2-amplified LUAD are related to its response to ICI therapy remains unclear. *HER2* amplification and mutations occur in approximately 2%–10% of NSCLCs, and effective treatment for this subset of LUAD has not been thoroughly investigated. RNA sequencing analysis indicated significantly higher PD-L1 expression at the mRNA and protein levels in patients with HER2-amplified LUAD than in those with breast invasive carcinoma (BRCA) and stomach adenocarcinoma (STAD). Patients with HER2-amplified LUAD also exhibited higher levels of tissue/blood TMB than the other two groups (Wang et al., 2022b). Moreover, the HER2-amplified LUAD group displayed an inflamed TIME characterized by higher levels of genes expressing classical MHC class I/II antigens, TAP1 and B2M (antigen processing machinery proteins), CD3, co-stimulatory molecules (such as CD28 and ICOS), cytotoxic effect-related genes (CD8A, IFNG, and GZMA), increased infiltration of CD4^+^ and CD8^+^ T cells, and M1 MQ, while showing lower infiltration of Tregs. IHC analysis further confirmed increased CD8^+^ TIL density and decreased FOXP3^+^ TIL densities in HER2-amplified LUAD specimens. In summary, patients with HER2-amplified LUAD exhibited higher immunogenicity, suggesting that they may benefit from ICI therapy. These patients also demonstrated a more favorable TIME, potentially making them better recognized by the immune system, thereby improving therapeutic efficacy in cancer immunotherapy. However, these studies were limited by the low frequency of HER2 amplification and mutations in patients with NSCLC; therefore, more comprehensive research is required to validate these observations (Wang et al., 2022b).

Some studies have suggested that the effectiveness of immunotherapy may be associated with the expression level of PD-1. *In silico* studies have categorized patients with lung squamous cell carcinoma into high and low PD-1 expression groups. Significant differences were observed in immune regulation pathways, particularly those associated with T cell activity. In the PD-1 high expression group, the most upregulated pathways, such as the MHC protein complex, were related to the immune system. TMB was high in both high and low PD-1 expression groups; however, no significant difference was observed between them. Owing to the lack of sequencing data for patients undergoing immunotherapy, determining whether these differences were related to the effectiveness of immunotherapy was impossible. However, differential expression of these genes may be a potential predictor of immunotherapy efficacy (Hu et al., 2020).

In summary, investigating the immunogenetic features of lung cancer may help identify potential candidates who can receive the maximum benefit from immunotherapy. Table 3 summarizes the *PD-1*, *PD-L1*, and *CTLA-4* polymorphisms associated with response to immunotherapy in patients with lung cancer.

**TABLE 3:** Identified polymorphisms in *PD-1*, *PD-L1*, and *CTLA-4* genes associated with clinical outcome of lung cancer patients.

Gene	Population	Polymorphisms associated with response to immunotherapy in lung cancer	Result	References
*CTLA-4 PD-1* *PD-L1*	166 NSCLC patients	-No correlation between SNPs and clinical outcome considering the entire cohort -Association between *PD-L1* rs4143815 SNP and the long clinical benefit group	*PD-L1* SNPs may be involved in predicting clinical outcome of NSCLC treated with ICI.	Minari et al. (2022)
*CTLA-4 PD-1* *PD-L1*	379 NSCLC patients	-Association of *PD-L1* rs2297136T > C with both better chemotherapy response and overall survival -Association of the *PD-L1* rs4143815C > G with better response to chemotherapy	Particular *PD-L1* polymorphisms may be useful for the prediction of clinical outcome of patients with advanced stage NSCLC after 1st line paclitaxel-cisplatin chemotherapy	Lee et al. (2016)
*PD-L1*	133 patients treated with nivolumab and 96 patients with no treatment history of ICI	-Association of *PD-L1* rs822339 and rs1411262 with overall survival in patients treated with nivolumab -Longer overall survival in patients with the A/A genotype of rs822339 than those with A/G or G/G genotypes - Longer overall survival in patients treated with nivolumab having the T/T genotype of rs1411262 compared to those with the C/T or C/C genotypes	Particular *PD-L1* polymorphisms could be used as potential prognostic markers in patients treated with nivolumab	Yoshida et al. (2021)
*PD-L1*	354 patients with early-stage NSCLC	-Association of the *PD-L1* rs4143815C>G, rs822336G>C, and rs822337T>A with worse survival outcomes -Decreased OS in a dose-dependent manner in case of combining the three SNPs, due to increase in the number of bad genotypes -Better survival of patients with a higher expression of PD-L1 mRNA	*PD-L1* polymorphisms may be useful for the prediction of prognosis in patients with surgically resected NSCLC.	Lee et al. (2017)
*KRAS*	219 patients with LUAD (108 with mutant *KRAS* and 111 with WT *KRAS*)	-Higher expression of PD-L1 in tumors harboring mutant *KRAS*-G12 V -Improved overall survival in KRAS mutant patients with higher expression of PD-L1 in tumor cells -Correlation between increased PD-L1 expression in immune cells and poor overall survival of *KRAS*-WT patients	-*KRAS* mutational status is associated with survival of LADC patients -Particular *KRAS* variants should be considered for immunotherapies	Falk et al. (2018)

NSCLC, Non-small cell lung cancer; CTLA-4, Cytotoxic T lymphocyte antigen 4; SNP, Single nucleotide polymorphism; PD-1, Programmed Cell Death Protein 1; LUAD, lung adenocarcinoma; PD-L1, Programmed death-ligand 1; KRAS, Kristen rat sarcoma viral oncogene homolog; ICI, Immune checkpoint inhibitor.

## 7 Conclusion

In conclusion, investigating the cellular and molecular backgrounds of immune cells infiltrating the lung TME can help predict the development and progression of lung cancer. Numerous efforts have been directed towards recognizing the genomic landscape to identify patients who are most responsive to specific immunotherapies. This holds the potential for early intervention and reducing the morbidity and mortality rates. scRNA-seq has emerged as a powerful technique that enables a deeper understanding of immune cell heterogeneity through transcriptomic analysis rather than surface markers, which are widely used in routine flow cytometry methods [9]. Future studies should prioritize large-scale genetic polymorphism analyses of different immune system compartments to determine their association with lung cancer progression, which could facilitate the development of individualized therapies. Given the significant variability in genotype frequencies and allele distributions among populations, more studies are required to identify population-specific and general variants associated with risk, clinical outcomes, and response to lung cancer treatment.
